# Serum brain-derived neurotrophic factor predicting reduction in pulse pressure after a one-hour rest in nurses working night shifts

**DOI:** 10.1038/s41598-018-23791-8

**Published:** 2018-04-03

**Authors:** I-Te Lee, Wayne Huey-Herng Sheu, Wen-Jane Lee, Der-Yuan Chen

**Affiliations:** 1 Division of Endocrinology and Metabolism, Department of Internal Medicine, Taichung Veterans General Hospital, Taichung, Taiwan; 2 School of Medicine, National Yang-Ming University, Taipei, Taiwan; 3 School of Medicine, Chung Shan Medical University, Taichung, Taiwan; 4 Department of Medical Research, Taichung Veterans General Hospital, Taichung, Taiwan; 5 Department of Internal Medicine, Taichung Veterans General Hospital, Taichung, Taiwan; 6 PhD Program in Translational Medicine and Rong Hsing Research Center for Translational Medicine, National Chung Hsing University, Taichung, Taiwan

## Abstract

Night shift work is associated with cardiovascular disease and central nervous system disorders in female nurses. Brain-derived neurotrophic factor (BDNF) exerts protective effects on neural and endothelial functions. This study examined the association between serum BDNF levels and pulse pressure after rest in female nurses working night shifts. In this study, blood samples were collected for BDNF measurement after a night shift when nurses had been working night shifts for three continuous weeks. Blood pressure was assessed before and after a one-hour morning rest within a week of resuming the night shift after one month without any night shift work. The pulse pressure of nurses (n = 48, age 29 ± 5 years) was significantly reduced (from 43 ± 7 to 41 ± 6 mmHg, P = 0.003) after rest, and serum BDNF were significantly and inversely correlated with pulse pressure changes (*r* = −0.435, P = 0.002). Higher serum BDNF was an independent factor for greater reduction in pulse pressure (95%CI = −0.609 ‒ −0.174, P = 0.001). Using a receiver operating characteristic curve analysis, serum BDNF >20.6 ng/mL predicted a pulse pressure reduction after a one-hour rest (sensitivity 66.7%, specificity 77.8%). In conclusion, higher serum BDNF predicted greater recovery of pulse pressure after a one-hour rest in female nurses after night shift work.

## Introduction

Night shift work is inevitable for nurses caring for inpatients. Pooled data from the Nurses’ Health Study (NHS) I and NHS II show that approximately 60% of female nurses had experienced night shift work^1^. According to the data from 39 hospitals in Taiwan, approximately 40% of female nurses had performed night shifts for more than 10% of their total work time^2^. In other reports from the NHS I, night shift work was significantly associated with coronary heart disease and ischemic stroke in female nurses^3,4^. Night shift work was recently reported to be significantly associated with all-cause mortality and cardiovascular mortality in female nurses who were followed for 22 years^5^.

Rotating shift work is also associated with the central nervous system disorders^6,7^. In one mouse study, the disturbance of circadian rhythms induced by exposure to light at night caused a depressive response^8^. Brain-derived neurotrophic factor (BDNF), a member of the neurotrophin family, plays important roles in protecting neurons and synaptic activity^9^. A reduction in circulating BDNF level is associated with depressive symptoms^10–12^, and serum BDNF levels can reflect an improvement in depressive symptoms after lifestyle interventions^13,14^ or pharmacological treatments^15,16^.

Notably, a low serum BDNF level is associated with forearm vascular resistance^17^. In one recent cross-sectional study, lower circulating BDNF concentrations were reported to be associated with coronary artery disease^18^; in a prospective study, a lower BDNF concentration was reported to predict coronary heart disease and higher all-cause mortality^19^. Therefore, insufficient BDNF levels may play an important role in linking central nervous system disorders and arterial stiffness^20^.

Pulse pressure, the difference between systolic and diastolic blood pressures, can reflect the severity of arterial stiffening^21^, and a higher pulse pressure predicts a higher cardiovascular mortality rate^22,23^. According to studies that stratified by age, pulse pressure is a more powerful predictor of cardiovascular mortality in young subjects than in older subjects^24,25^. Furthermore, long-term reduction in pulse pressure is significantly associated with survival^26^. A short-term reduction in pulse pressure from peak exercise to rest is predictive of survival in subjects with normal myocardial perfusion imaging^27^, or after hemodialysis in patients with end-stage renal disease^28^.

Based on psychological stress, delayed recovery of systolic blood pressure after 45 minutes might predict the carotid intima-media thickness and hypertension^29,30^. According to Mathiassen *et al*.^31^ a delayed recovery of blood pressure increased by physical work was observed after a one-hour rest period. In the present study, we examined the relationship between serum BDNF levels and changes in pulse pressure after a one-hour rest period in female nurses working night shifts.

## Methods

### Subjects

This prospective observational study was conducted at Taichung Veterans General Hospital, Taiwan. The included subjects were female nursing staff members aged ≥20 years who worked in wards with a monthly rotating shift pattern. The exclusion criteria were (1) working night shifts at the time of the screening, (2) known diabetes mellitus, (3) established cardiovascular diseases, (4) current severe infection or inflammation, (5) a history of malignancy, (6) a history of psychological or mood disorder, (7) a history of substance or alcohol abuse, and (8) post-menopause or pregnancy. The study complied with the Declaration of Helsinki. All experimental protocols were approved by the Institutional Review Board of Taichung Veterans General Hospital and the written informed consent was obtained from all subjects. The methods were carried out in accordance with the approved guidelines.

### Study procedures

The aim of this study was to determine whether serum BDNF levels in female nurses who had worked a stable night shift predicted pulse pressure after a one-hour rest period when they rotated to the next night shift. The study subjects were screened when they were just before they rotated to night shift work for the following month. During the first night shift month, a three-week observation period confirmed that the subjects were performing their night shifts in a stable pattern. In the fourth week, the subjects were scheduled for a morning appointment after overnight fasting. Blood samples were collected to measure fasting glucose, insulin, lipid profile, serum creatinine, C-reactive protein (CRP) and BDNF levels after anthropometric measurements were obtained.

After the first night shift month, the subjects underwent a wash-out period during which they did not perform night shift work for a month. The participants were scheduled for another morning visit during the first week of their rotation to a second month of night shift work after the one-month wash-out period. During this visit, blood pressure was measured at the right brachial artery, and the mean of two separate measurements with a interval of 1 min was recorded after the subjects had rested in a lying position for 10 minutes in the morning following the end of the night shift. Blood samples were not collected during this visit to avoid potential impacts of any invasive procedures on the blood pressure measurements. Then, the subjects were asked to take a nap on the same bed for one hour. After the nap, blood pressure was measured again.

### Biochemical analyses

Glucose levels were determined using an oxidase‒peroxidase method (Wako Diagnostics, Tokyo, Japan), and insulin levels were determined using a commercial immunoassay (Roche Diagnostics, Indianapolis, USA). Serum levels of creatinine and lipids were measured using commercial kits (Beckman Coulter, Fullerton, USA), and CRP levels were measured using an immunochemical assay with purified Duck IgY (∆Fc) antibodies (Good Biotech Corp., Taichung, Taiwan). Serum human BDNF levels was measured using an enzyme immunoassay method (R&D Systems, Minneapolis, USA). The intra‒ and inter‒ assay coefficients of variation for BDNF measurements were 4.1% and 9.0%, respectively. The sensitivity of the BDNF measurement was 0.02 ng/mL. The formula [fasting insulin (μU/mL) × fasting glucose (mmol/L)]/22.5 was used to calculate the homeostasis model assessment for insulin resistance (HOMA-IR) index^32^. The estimated glomerular filtration rate (eGFR) was calculated as 186 × [serum creatinine (mg/dL)]^−1.154^ × [age (years)]^−0.203^ × 0.742 based on the Modification of Diet in Renal Disease (MDRD) equation^33^.

### Statistical analysis

Continuous variables are presented as the means ± standard deviations (SD). An independent samples *t*-test was used to detect significant differences between two groups. A paired *t*-test was used to detect significant differences in blood pressure before and after the one-hour rest. The correlation between serum BDNF levels and changes in the pulse pressure was determined using Pearson’s correlation coefficient. A multivariate linear regression analysis was used to assess the association between the changes in pulse pressure and serum BDNF levels. Statistical analyses were performed using SPSS version 22.0 software (International Business Machines Corp, New York, USA).

## Results

**Figure 1:**
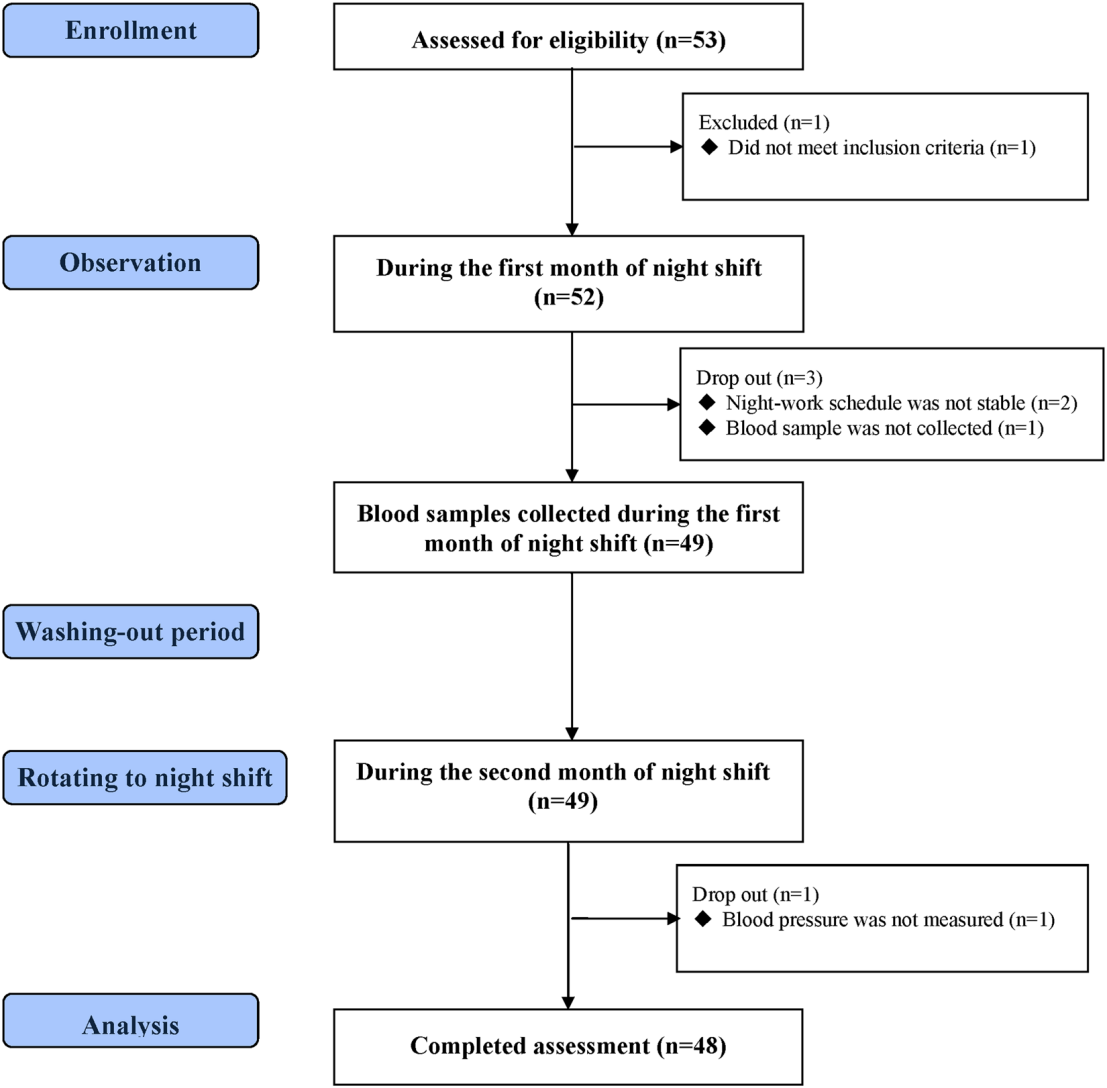
Flow diagram of the enrollment of study subjects.

**Figure 2:**
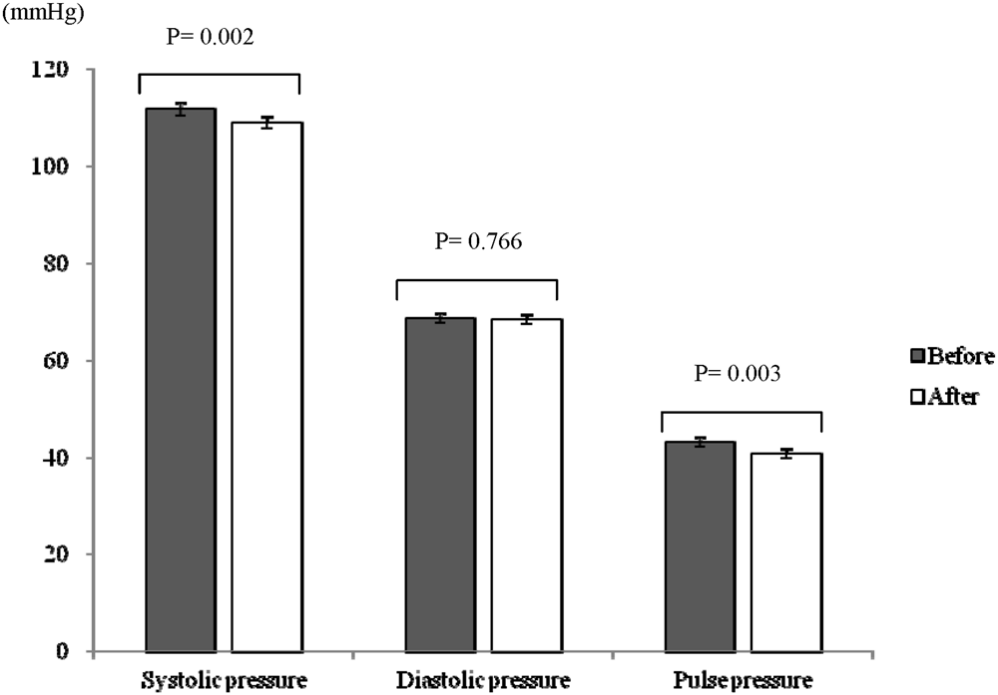
Blood pressure measured before and after a one-hour rest in subjects within a week of starting a new period of night-shift work.

**Figure 3:**
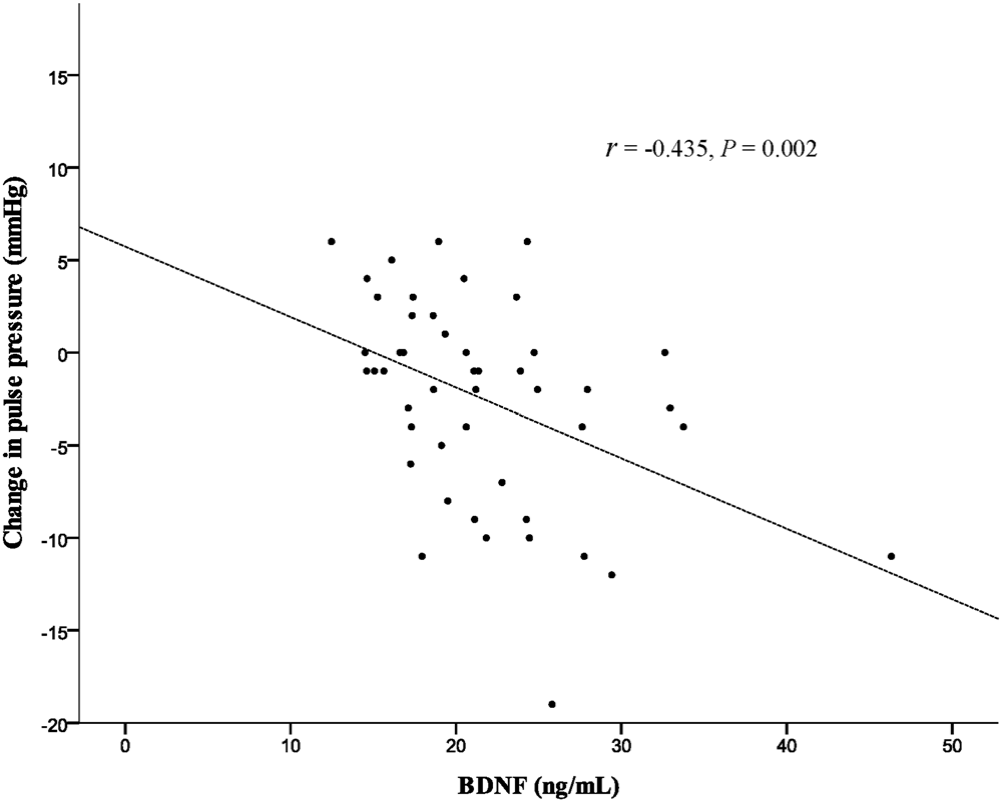
A significant inverse correlation is demonstrated between serum BDNF levels and changes in pulse pressure (*r* = correlation coefficient).

A total of 53 female nurses were screened, of whom 48 completed the study assessments (Fig. 1). Table 1 shows the baseline clinical characteristics of the study subjects during the first night shift month, measured after they had performed night shift work for three weeks. The mean age of the subjects was 29 ± 5 years, and the mean serum BDNF was 21.6 ± 6.2 ng/mL. After rotating back to night shifts following a wash-out month, the subjects showed a significant reduction in the mean systolic pressure (112 ± 9 mmHg vs. 109 ± 9 mmHg, P = 0.002) after a one-hour rest period but not a significant change in the mean diastolic pressure (69 ± 7 mmHg vs. 68 ± 7 mmHg, P = 0.766). Pulse pressure (43 ± 7 mmHg vs. 41 ± 6 mmHg, P = 0.003) was also significantly reduced after rest (Fig. 2). Serum BDNF levels during the first night shift month were significantly and inversely correlated with the change in pulse pressure (correlation coefficient of −0.435, P = 0.002; Fig. 3).

**Table 1:** Clinical data of subjects (n = 48) at baseline.

	Mean ± SD
Age (year)	29 ± 5
BMI (kg/m^2^)	22.5 ± 3.6
Waist circumference (cm)	76.2 ± 11.1
Fasting glucose (mmol/L)	4.5 ± 0.5
Fasting insulin (µIU/mL)	7.2 ± 4.9
HOMA-IR	1.5 ± 1.1
Total cholesterol (mmol/L)	4.4 ± 0.6
HDL cholesterol (mmol/L)	1.7 ± 0.4
Triglyceride (mmol/L)	0.7 ± 0.4
AST (U/L)	18 ± 6
ALT (U/L)	14 ± 8
eGFR (mL/min/1.73 m^2^)	118 ± 18
TSH (μU/mL)	1.5 ± 0.8
CRP (mg/L)	1.1 ± 1.8
BDNF (ng/mL)	21.6 ± 6.2
Work experience (month)	71 ± 62
Night-shift work experience (month)*	63 ± 62
Frequency of alcohol consumption	
<1 time/month, N (%)	26 (54.2%)
≥1 time/month and <1 time/week, N (%)	17 (35.4%)
≥1 time/week, N (%)	5 (10.4%)
Frequency of coffee consumption	
<1 time/day, N (%)	28 (58.3%)
≥1 time/day, N (%)	20 (41.7%)
Smoking history, N (%)	0 (0%)
Use of sedatives in the past 3 months, N (%)	2 (4.2%)
Use of anti-hypertensive drugs in the past 3 months, N (%)	0 (0%)
Exercise	
<150 min/week, N (%)	46 (95.8%)
≥150 min/week, N (%)	2 (4.2%)

AST = aspartate aminotransferase, ATL = alanine aminotransferase, BDNF = brain-derived neurotrophic factor, BMI = body mass index, CRP = C-reactive protein, eGFR = estimated glomerular filtration rate, HDL = high-density lipoprotein, HOMA-IR = homeostasis model assessment of insulin resistance, TSH = thyroid stimulating hormone.

*Night-shift work experience refers to the total work experience after having worked the night shift.

According to the changes in pulse pressure after a one-hour rest, we divided the subjects into a pulse-pressure (P-P) reduction group (n = 30) and a no P-P reduction group (n = 18). Table 2 shows that serum BDNF was significantly higher in the P-P reduction group than in the no P-P reduction group (23.1 ± 6.6 ng/mL vs. 19.1 ± 4.8 ng/mL, P = 0.032). Pre-rest diastolic pressure in the P-P reduction group was significantly lower than in the no P-P reduction group (67 ± 5 mmHg vs. 71 ± 8 mmHg, P = 0.017). Multivariate linear regression analyses performed after adjustment for age, body mass index and pre-rest diastolic blood pressure showed that higher serum BDNF was significantly associated with a greater reduction in pulse pressure after a one-hour rest (linear regression coefficient of −0.391, 95% confidence interval (CI) between −0.609 and −0.174; P = 0.001; Table 3).

**Table 2:** The characteristics of subjects grouped according to pulse-pressure (P‒P) change.

	P‒P reduction (n = 30)	no P‒P reduction (n = 18)	P
**At baseline**			
Age (year)	29 ± 5	29 ± 6	0.935
BMI (kg/m^2^)	22.1 ± 3.6	23.1 ± 3.8	0.383
Waist circumference (cm)	76.0 ± 11.3	76.6 ± 11.1	0.862
Fasting glucose (mmol/L)	4.5 ± 0.6	4.5 ± 0.4	0.666
HOMA-IR	1.6 ± 1.2	1.3 ± 0.6	0.368
Total cholesterol (mmol/L)	4.4 ± 0.6	4.2 ± 0.6	0.277
HDL cholesterol (mmol/L)	1.7 ± 0.4	1.6 ± 0.3	0.605
Triglyceride (mmol/L)	0.7 ± 0.4	0.6 ± 0.4	0.606
AST (U/L)	19 ± 8	17 ± 4	0.466
ALT (U/L)	14 ± 9	15 ± 7	0.862
eGFR (mL/min/1.73 m^2^)	119 ± 19	115 ± 17	0.465
TSH (μU/mL)	1.5 ± 0.8	1.5 ± 0.7	0.926
CRP (mg/L)*	1.1 ± 1.6	1.3 ± 2.0	0.482
BDNF (ng/mL)	23.1 ± 6.6	19.1 ± 4.8	**0.032**
Work experience			
Total (month)	65 ± 54	81 ± 75	0.402
Night shift (month)^#^	58 ± 55	70 ± 74	0.527
Frequency of alcohol consumption			0.454
<1 time/month, N (%)	18(60.0%)	8(44.4%)	
≥1 time/month, N (%)	12(40.0%)	10(55.6%)	
Frequency of coffee consumption			0.545
<1 time/day, N (%)	19(63.0%)	9(50.0%)	
≥1 time/day, N (%)	11(36.7%)	9(50.0%)	
**Rotating to night shift after wash-out period**			
Blood pressure before rest			
Systolic pressure (mmHg)	111 ± 8	113 ± 11	0.666
Diastolic pressure (mmHg)	67 ± 5	71 ± 8	**0.017**
Pulse pressure (mmHg)	45 ± 7	41 ± 6	0.082
Blood pressure after rest			
Systolic pressure (mmHg)	108 ± 9	111 ± 9	0.178
Diastolic pressure (mmHg)	69 ± 6	68 ± 7	0.635
Pulse pressure (mmHg)	39 ± 6	44 ± 6	**0.013**
Heart rate			
Before rest (min^−1^)	70 ± 10	71 ± 11	0.775
After rest (min^−1^)	69 ± 11	68 ± 10	0.611

ATL = alanine aminotransferase, AST = aspartate aminotransferase, BDNF = brain-derived neurotrophic factor, BMI = body mass index, eGFR = estimated glomerular filtration rate, HDL = high-density lipoprotein, HOMA-IR = homeostasis model assessment of insulin resistance, P‒P reduction = pulse‒pressure reduction, TSH = thyroid stimulating hormone.

*CRP was logarithm-transformed (log) in analyses due to skewed distribution.

^#^Night-shift work experience refers to the total work experience after having worked the night shift.

**Table 3:** Multivariate regression analysis showing an independent association between serum BDNF and pulse-pressure change.

	Crude			Model 1			Model 2		
	B	95%CI	P	B	95%CI	P	B	95%CI	P
BDNF (ng/mL)	−0.381	(−0.615, −0.147)	**0.002**	−0.375	(−0.611, −0.140)	**0.002**	−0.391	(−0.609, −0.174)	**0.001**
Age (years)				0.197	(−0.102, 0.496)	0.190	0.219	(−0.057, 0.495)	0.118
BMI (kg/m^2^)				0.012	(−0.393, 0.417)	0.953	−0.285	(−0.710, 0.140)	0.183
Diastolic blood pressure (mmHg)							0.343	(0.109, 0.576)	**0.005**

B = linear regression coefficient, BDNF = brain-derived neurotrophic factor, BMI = body mass index, CI = confidence interval.

Model 1: adjusted for age and BMI; model 2: adjusted for age, BMI and diastolic blood pressure before taking a nap.

**Figure 4:**
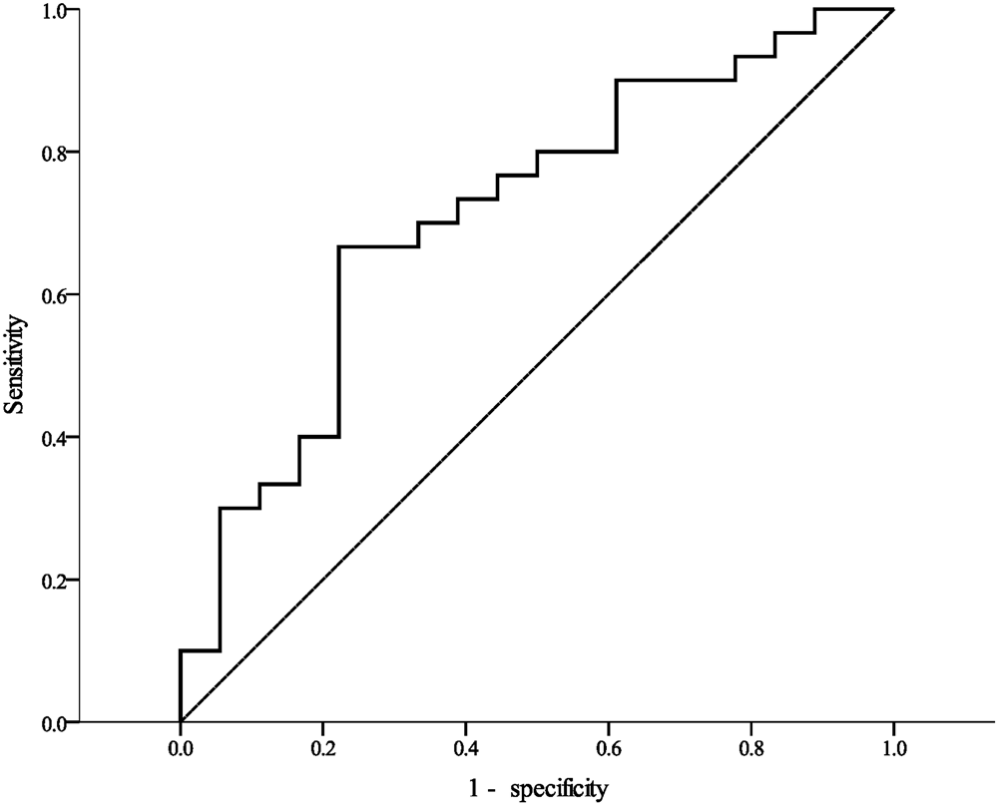
Receiver operating characteristic (ROC) analysis curve for serum BDNF level to differentiate a reduction in pulse pressure after a one-hour rest (area under the curve 0.715, 95%CI = 0.563–0.867).

Based on the receiver operating characteristic (ROC) curve analysis, a cut-off value of 20.6 ng/mL for serum BDNF levels provided a sensitivity of 66.7% and specificity of 77.8% for differentiating a reduction in pulse pressure after a one-hour rest in nurses performing night shift work (Fig. 4).

## Discussion

The main finding in our study was that higher levels of serum BDNF levels in female nurses during night shift work predicted a greater reduction in pulse pressure after a short morning rest when rotating back to night shift work. Arterial stiffening describes the loss of the arterial wall’s elastic capacity to buffer the blood flow from ventricular ejection^34^. Widening pulse pressure, resulting from a surge of systolic pressure against a non-compromised arterial wall followed by a low diastolic pressure, reflects the severity of arterial stiffness^35^. Arterial stiffness may involve not only the extracellular destruction of the vessel wall^36,37^, but also dysfunction of vascular smooth muscle cells^38^. Tropomyosin-related kinase receptor B (TrKB), a high-affinity receptor for BDNF, is expressed in the vascular smooth muscles^39,40^. Inactivation of the BDNF-TrkB signaling pathway in vascular smooth muscle cells may impair coronary vascular integrity and function^41^. Following treatment with empagliflozin, a sodium glucose co-transporter 2 inhibitor, higher BDNF expression was associated with the reduction of coronary arterial fibrosis in a diabetic mouse model^42^.

Night shift work interrupts the circadian rhythm and may impair vascular functions controlled by autonomic activity^43^. Increased arterial stiffness with decreased vascular activity, estimated using the reactive hyperemia index, has been found in subjects currently performing night shift work when compared with those not currently working night shifts^44^. Although the high blood pressure experienced during the work period normally decreases after a rest period, a delayed recovery of the high blood pressure during rest periods is more common in female nurses working night shifts than in those working day shifts^45^. High blood pressure may be the result of circadian disruption when rotating to night shift work. Kubo *et al*.^46^ also reported a reduction in the coronary flow reserve of the left anterior descending artery in female nurses the morning after working a night shift.

Regular coffee consumption increases BDNF expression in the in the mouse hippocampus^47^; however, the change in circulating BDNF was not reported. In the present study, coffee-consumption frequency did not significantly affect the serum BDNF (21.5 ± 5.3 ng/mL in the subjects who consumed coffee <1 time per month vs. 21.7 ± 7.5 ng/mL in the subjects who consumed coffee ≥1 time per month). Although the intake of a single-dose of coffee fruit extract was also reported to increase the circulating BDNF levels of healthy subjects within 120 min^48^, serum BDNF was assessed in a fasting state in the present study. This may explain why coffee consumption was not associated with serum BDNF levels in the present study. In contrast, it has been reported that arterial stiffness might be induced by either acute or chronic coffee consumption^49–51^. Souza *et al*.^52^ also reported that a single dose of coffee might increase post-exercise systolic blood pressure. The impact of coffee consumption on pulse pressure recovery after rest following a night shift should be further investigated.

We collected serum BDNF data in the morning after a night shift when the participants had worked the night shift for three weeks, and we measured the morning blood pressure after a night shift in female nurses who had rotated to a new series of night shifts within one week of the rotation. We found that high morning serum BDNF levels during stable night shift work could predict the quick recovery of a widening pulse pressure after a one-hour rest in the female nurses who were beginning a new night-shift schedule. However, the present study had certain limitations. First, the study only included young female nurses. Despite the delayed recovery of blood pressure in men performing night shift work^53^, the impact of shift work on mental health has been reported to differ between genders^54^. Therefore, our findings in female nurses cannot be applied men. In addition, our findings cannot be applied to postmenopausal women since a lower circulating BDNF concentration has been observed in postmenopausal females than in young females^55,56^. Second, menstrual cycle information was not recorded for analysis in the present study. An increase in circulating BDNF during the luteal phase compared with the follicular phase has been reported in woman with normal menstrual cycles^56,57^. Additionally, Dunne *et al*.^58^ reported lower blood pressure during the luteal phase than during the in follicular phase, and Mills *et al*.^59^ reported a difference in blood-pressure recovery after stress between the luteal phase and follicular phase. Therefore, the menstrual cycle could be a confounding factor in studies of young females. Third, we did not measure the serum BDNF levels when blood pressure was assessed because of the relationship between BDNF and vascular functions consistently reported in previous studies^39–41,60–62^. Fourth, the change in diastolic blood pressure was not statistically significant after rest. We did not further examine the underlying causes of differences in recovery between systolic and diastolic blood pressures. Finally, we did not explore the mechanism linking BDNF and pulse-pressure recovery after night shift work; moreover, we did not assess the long-term cardiovascular outcomes in subjects with high pulse pressures after a one-hour rest period.

In conclusion, the serum BDNF concentration measured during a stable period of night shift work predicted a reduction in pulse pressure in female nurses after one hour of rest when they were rotating to a new period of night shifts. These findings may indicate a possible modulatory effect of circulating BDNF on the recovery of pulse pressure in female nurses after they have worked rotating night shifts. Further studies are necessary to investigate the underlying mechanisms linking serum BDNF during night shift work to the long-term cardiovascular prognosis.
